# Knee-Sparing Resection and Reconstruction Surgery for Bone Sarcoma Using 3D-Surgical Approach: Average of 5-Year Follow-Up

**DOI:** 10.3390/medicina61030476

**Published:** 2025-03-08

**Authors:** Amit Benady, Noy Yehiel, Ortal Segal, Omri Merose, Amir Sterenheim, Osnat Sher, Ben Efrima, Eran Golden, Yair Gortzak, Solomon Dadia

**Affiliations:** 1 Levin Center for 3D Printing and Surgical Innovation, Tel Aviv Sourasky Medical Center, Tel Aviv 6423906, Israel; noy.yehiel@mail.huji.ac.il (N.Y.); erangol@tlvmc.gov.il (E.G.); dadias@tlvmc.gov.il (S.D.); 2 Sackler School of Medicine, Tel Aviv University, Tel Aviv 69978, Israel; ortalsegalmd@gmail.com (O.S.); omrim@tlvmc.gov.il (O.M.); amirsternheim@gmail.com (A.S.); osnats@tlvmc.gov.il (O.S.); benefrima@gmail.com (B.E.); yairg@tlvmc.gov.il (Y.G.); 3 Division of Orthopedic Surgery, Tel Aviv Sourasky Medical Center, Tel Aviv 6423906, Israel; 4 Faculty of Medicine, Hebrew University of Jerusalem, Jerusalem 9190501, Israel; 5 National Unit of Orthopedic Oncology, Tel Aviv Sourasky Medical Center, Tel Aviv 6423906, Israel; 6 Department of Pathology, Tel Aviv Medical Center, Tel Aviv 6423906, Israel

**Keywords:** bone sarcoma, knee-sparing surgery, 3D workflow, 3D printing, patient-specific instruments (PSIs)

## Abstract

*Background and Objectives*: To date, the gold standard of care for bone sarcomas is limb salvage surgical resection. In cases where the tumor arises in the distal femur or proximal tibia near the joint line, knee-sacrificing surgery is typically performed, followed by reconstruction with oncological megaprostheses. This study aims to evaluate the effectiveness of a precise 3D-based surgical approach for knee-sparing tumor resections, assessing its feasibility and its impact on surgical, oncological, and functional outcomes. *Materials and Methods*: This single-center retrospective study presents the surgical and oncological outcomes of knee-sparing surgeries following bone sarcoma resections. All patients underwent either intercalary or geographic resection, and reconstruction was tailored to each patient, using either an allograft or a titanium alloy Ti64 implant, depending on the specific requirements of the case. *Results*: A total of 23 patients (average age 21.04 years, 14 males) were included, with an average postoperative follow-up of 58 months (range: 12–102 months). Clear surgical margins were achieved in all patients, with 16 patients (69.5%) showing wide negative margins (R0) and the rest showing close negative margins (R1). Resections were primarily intercalary (17 patients, 73.9%), with 6 patients (26.1%) undergoing geographic resections. Reconstruction methods included allografts (9 patients, 39.3%), vascularized fibula and allograft (8 patients, 34.7%), and printed Ti64 cage reconstructions (6 patients, 26.0%). At the last follow-up, 19 patients (82.6%) were disease-free, 3 patients (13.4%) were alive with evidence of disease, and 1 patient (4%) was dead of disease. Complications included four cases of non-union that required revision surgery, as well as two local recurrences, which necessitated revision surgery to a modular endoprosthesis and above-knee amputation. The average MSTS at the final follow-up was 23.16 ± 5.91. *Conclusions*: The use of 3D-printed PSIs for knee-sparing bone tumor resections has emerged as the gold standard, enhancing both surgical and oncological outcomes. A future challenge lies in improving reconstruction techniques, shifting from traditional allografts to customized Ti64 printed lattice implants. As personalized healthcare and additive manufacturing continue to advance, the future of orthopedic oncology will likely see more precise, durable, and biologically integrated implants, further improving patient outcomes.

## 1. Introduction

Osteosarcoma and Ewing’s sarcoma account for 15% of all cancerous malignancies in patients between the ages of 5–25 [1,2]. Overall, the distal femur is the most prevalent site for primary bone cancers, followed by the proximal tibia [3] and the metaphysis [4]. Until the late 1960s, the treatment in such cases depended primarily on surgical amputation, and 5-year survival rates remained below 20%, with patients dying from lung metastasis. However, the introduction of effective chemotherapeutic agents and the advancements in neo-adjuvant chemotherapy over the past 50 years have led to significantly improved 5-year survival rates, now reaching 66–82% [5]. Surgical methods and reconstructive materials and technologies have made these patients candidates for limb salvage surgeries and have yielded successful and safe margin resection and reconstruction of a viable and functional extremity.

To date, metallic modular endoprostheses are commonly used following limb salvage surgeries and have high success rates, enabling early weight-bearing and the maintenance of joint mobility [6,7,8]. Mechanical obstacles and aseptic loosening have also decreased with the change from fixed-knee hinge joints to rotating-hinge knee joints due to tibial rotation reducing stress on the bone–implant interface and, thus, decreasing the loosening rate [9,10,11]. However, Sambri et al., in their systematic review, demonstrated that these implants continue to show higher rates of failure and complications compared to non-oncologic arthroplasty procedures, with a notably increased need for revision surgeries. The overall complication rate for megaprostheses was reported as 29.7%, with common issues including soft tissue failure and infection [12]. Deep infection rates for megaprostheses were found to range from 14.5 to 19.2%. Interestingly, the infection rates in traumatology patients using megaprostheses were lower compared to oncologic cases [13].

Joint-sparing procedures have become feasible thanks to the high precision achieved through three-dimensional (3D) pre-surgical planning and the use of intraoperative patient-specific instruments (PSIs). Historically, pre-surgical planning was based on 2D radiographs, with surgeons attempting to execute the plan using standard instruments. This approach often failed to account for the patient’s unique anatomy, leading to inaccuracies in reconstruction [14]. 3D-printed PSIs not only allow for accurate tumor resection, but also facilitate the harvesting of bone grafts from a bone bank that perfectly fits the required bone defect reconstruction. These days, accurate resection using PSI is the gold standard for tumor resections, including creating ideal allograft reconstructions. Looking ahead, the future lies in the use of 3D-printed titanium alloy Ti64 patient-specific implants, which promise even greater precision and personalization [15].

This study presents a surgical method that allows the resection of bone tumors surrounding the knee joint with a safe and accurate minimal margin from the joint surface, as well as the subsequent reconstruction of the bone gap with either a tailored allograft or a Ti64 customized implant. We describe our experience with this method during a four-year period and the future implications for patient care. This 3D approach not only allows surgeons to preserve the native knee joint and improve the patient’s future quality of life, but also reduces the complications that follow reconstruction with traditional metallic modular endoprostheses.

## 2. Materials and Methods

### 2.1. Patients

This retrospective study presents the postoperative outcomes of a cohort of patients who underwent knee-sparing surgery using a 3D surgical approach, including digital visualization and PSIs, between 2016-2023. All patients were invited for regular visits to assess local recurrence, metastases, and functional outcomes (see Table 1, Table 2 and Table 3 for full details). The exclusion criteria included tumors involving physis or epiphysis. In cases where the tumor invaded past the metaphysis, knee-sacrificing surgery was applied. Margins were categorized as positive (R2), negative with microscopic residual disease (R1, <1 mm), or negative with no residual disease (R0, ≥1 mm). Functional outcomes were evaluated using the MSTS93 (Musculoskeletal Tumor Society) scoring system at the final follow-up. The revised MSTS93 score includes six items tailored to the affected extremity. For the upper extremity, the evaluated items are pain, function, emotional acceptance, hand positioning, manual dexterity, and lifting ability. For the lower extremity, the items are pain, function, emotional acceptance, support, walking ability, and gait aid/gait. Each item is scored on a scale from 0 to 5, with a maximum total score of 30. The total score is then converted into a percentage to facilitate interpretation and comparison. This study was approved by the Tel Aviv Medical Center ethics committee.

### 2.2. Preoperative Planning and Simulation

**Figure 1:**
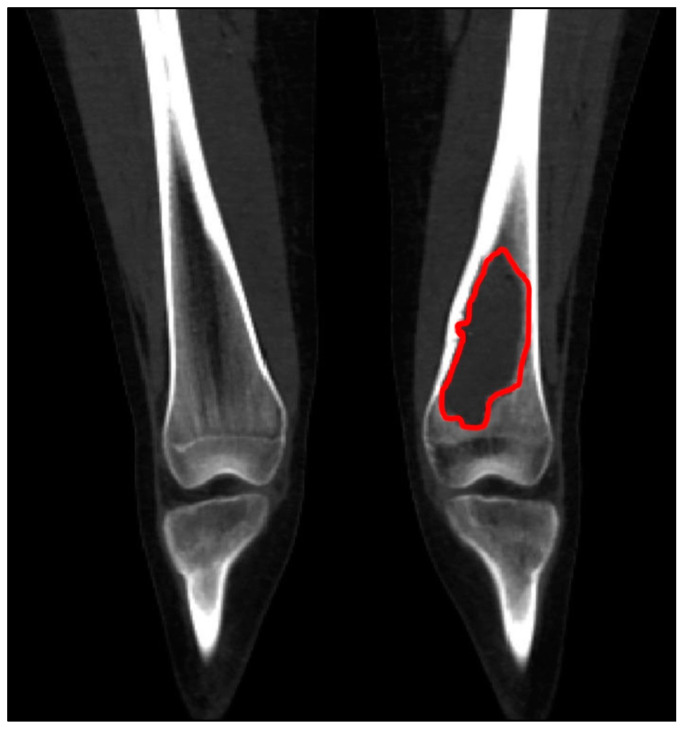
An example of an MRI demarcating the tumor margins (red line) superimposed over a CT scan demarcating the bone anatomy.

Initially, the surgeon provided a biomedical engineer with a computerized tomographic (CT) scan and a Magnetic Resonance Imaging (MRI) scan of the relevant lower limb. The CT scan slices were 0.5–1 mm thick to define the exact bone anatomy, while the MRI scan slices were 4 mm and defined the tumor and soft tissue borders. All the 2D images obtained from both modalities were imported into FDA-approved image-processing software (Mimics^®^, Materialise, N.V. Leuven, Belgium, or Intellispace Portal V9 and V11, Philips Healthcare, Best, Netherlands). The images were merged and segmented to produce a 3D digital model that contained the precise bone anatomy superimposed with the exact tumor borders (Figure 1). Then the model was exported as an STL file into FDA-approved CAD software (3-matic^®^, Materialise N.V. Leuven, Belgium). Following the completion of the 3D model, the surgeon and the engineer worked together to determine the surgical approach according to the specific tumor and clinical scenario.

### 2.3. 3D-Printed Patient-Specific Instruments

**Figure 2:**
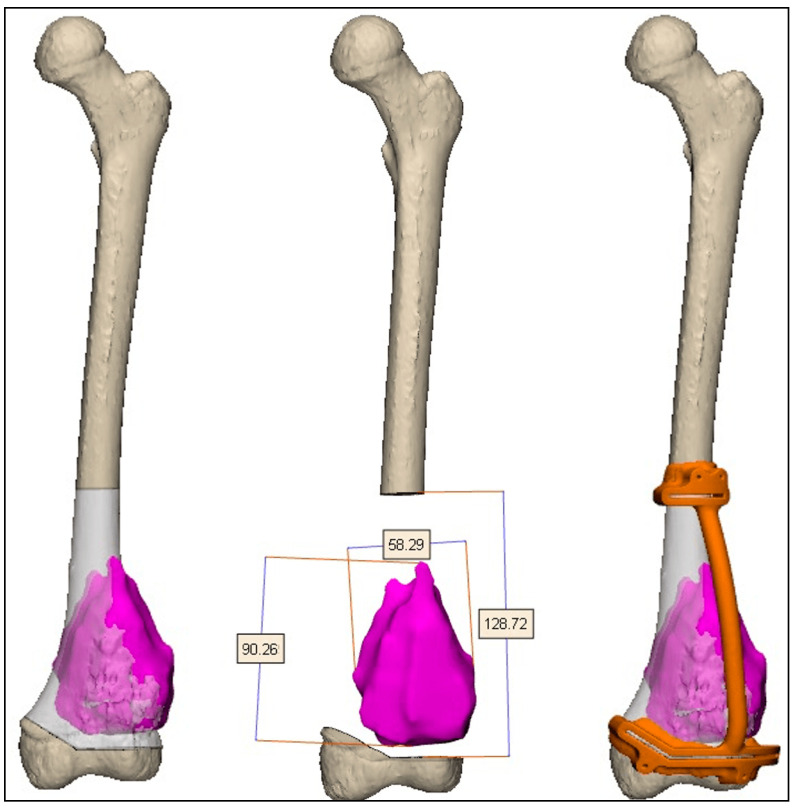
An example of surgical osteotomy planning over bone imaging of the distal femur. Note the proximity of the tumor to the knee joint.

**Figure 3:**
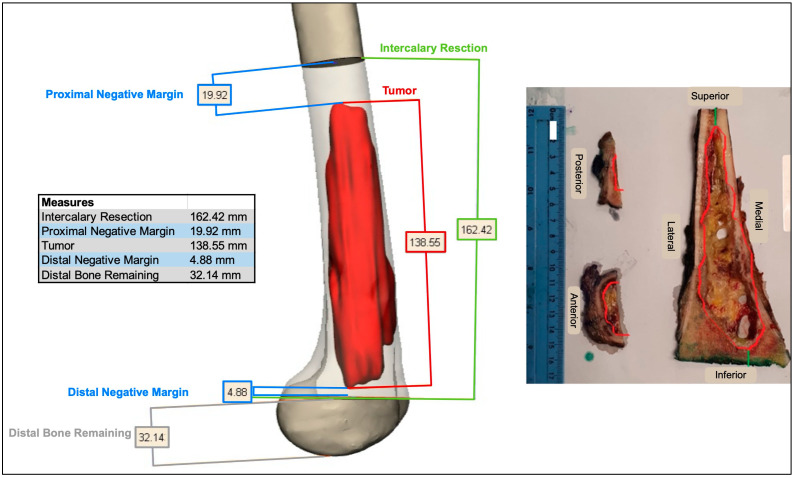
An example of the surgical planes superimposed over 3D bone imaging, with measurements of the distance between the osteotomy site and the tumor borders. On the right, a pathological specimen demonstrates the same margin post-tumor resection.

**Figure 4:**
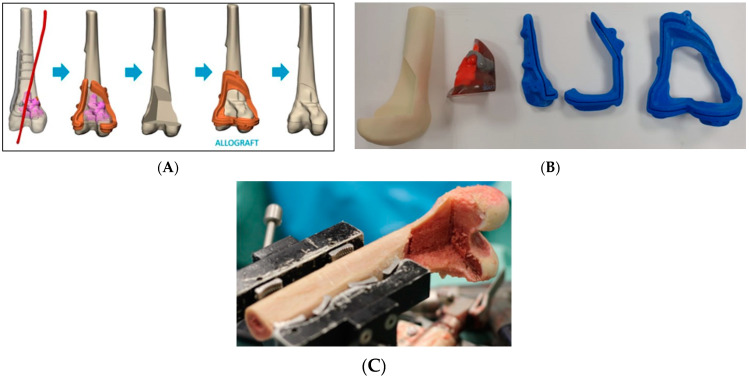
(**A**) A digital 3D model of an atypical cartilaginous tumor located in the distal femur. From left to right: the femur prior to surgery, PSI planning, resection, and allograft reconstruction. (**B**) 3D-printed anatomical models of the bone and PSIs. (**C**) The cadaveric bone used to prepare the allograft with the same PSI for a perfect reconstruction fit.

**Figure 5:**
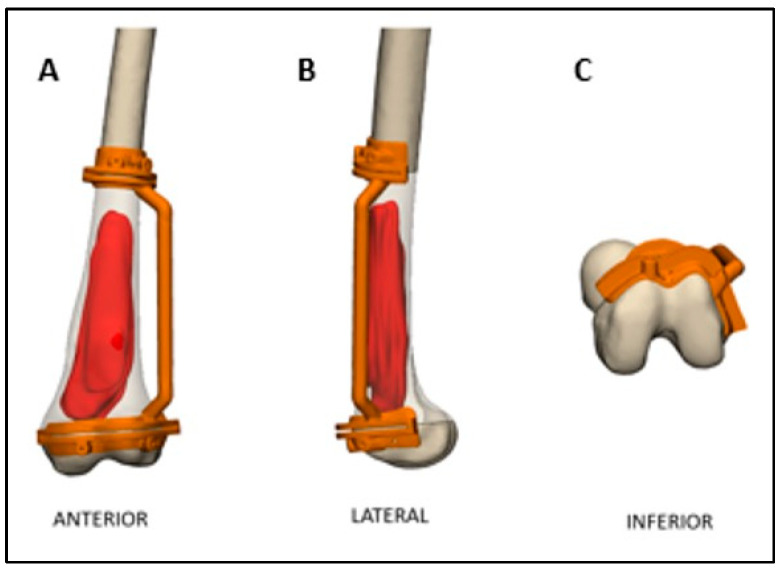
PSIs are planned with a patient-specific design according to their bone morphology in all 3 planes to ensure perfect placement and exact bone cuts.

The cutting planes were located on the bone 0.5-2 cm from the tumor margin. Cutting the plane adjacent to the joint line was more challenging due to the need to maintain a negative safe margin while staying at least 2 cm from the knee joint surface to spare the joint during the surgery. Notably, the resection plane could contain one or more osteotomies (Figure 2). These two planes determined the bone fragment that needed to be removed before executing the pre-surgical osteotomy plan (Figure 3). After the surgeon was satisfied with the pre-surgical plan, a surgical cutting guide (i.e., PSI) was designed based on the two cutting planes (Figure 4). This PSI provided accurate guidance for intraoperative osteotomies and was designed with a unique shape that follows the bone morphology of the patient to ensure exact placement, with a 1 mm thickness slit at each cutting plane and a handle located 3-5 cm away from the bone (Figure 5). After completing the surgical plan and creating digital models, a 1:1 anatomical model of the limb, including the tumor, was 3D-printed. The tumors were fabricated from VERO material using PolyJet technology (Stratasys, Eden Prairie, Minnesota; Rehovot, Israel) due to its rigid, opaque properties that provide excellent visual detail. The limb models were printed from ASA, while the PSI was reevaluated and approved by the surgeon, and then two cutting PSIs were printed from biocompatible, high-strength, and thermal-resistant material (ULTEM™ 1010) by a Fused Deposition Modeling (FDM) printer (Fortus 450 mc, Stratasys, Eden Prairie, Minnesota; Rehovot, Israel). Finally, the PSIs were washed and double-packed before undergoing a standard autoclave sterilization process in order to be brought into the surgical theatre. In addition, a similar PSI was printed and used for the preparation of the cadaveric allograft. In cases where a Ti64 implant was used for reconstruction, they were designed in-house using 3DXpert software (3D Systems, Rock Hill, South Carolina, USA), with lattice and solid structures to promote bone ingrowth, mechanical stability, and reduced stress shielding. All implants underwent finite element analysis (FEA) to predict potential failure and were designed based on the optimal design parameters. For specific cases, additional features like intramedullary nailing systems and screw holes for rotational stability were incorporated. The implants were manufactured using Direct Metal Printing (DMP) with Ti64 medical-grade powder (3D Systems, Tuvia Sharon, Israel). Post-processing involved heat treatment and dry electropolishing to reduce residual stresses that could cause cracks in the final parts.

## 3. Results

This study includes 23 patients (14 males and 9 females) with an average age of 21 years, ranging from 6 to 59 years. The most common diagnosis was osteosarcoma (OSA), affecting 14 patients (60.9%), followed by Ewing’s sarcoma in 5 patients (21.7%), chondrosarcoma in 3 patients (13%), and malignant fibrous histiocytoma (MFH) of bone in 1 patient (4.4%). The majority of tumors were located in the femur in 18 patients (78.2%), with the tibia involved in 5 cases (21.7%).

Resection types included 17 intercalary resections (73.9%) and 6 geographic resections (26.1%). Intercalary resections were most frequently performed for OSA (10 patients, 58.8%), followed by Ewing’s sarcoma (4 patients, 23.5%), chondrosarcoma (2 patients, 11.8%), and MFH (1 patient, 0.5%). Geographic resections were performed in cases of OSA (4 patients, 66.6%), Ewing’s sarcoma (1 patient, 16.6%), or chondrosarcoma (1 patient, 16.6%).

Regarding reconstruction methods, 9 patients (39.1%) received allografts, 8 patients received vascularized fibula and an allograft (34.7%), and 6 patients received a printed Ti64 cage (26.0%). Clear surgical margins were achieved in all patients, with 16 patients (69.5%) demonstrating wide negative margins (R0) and the remaining patients having close negative margins (R1). The follow-up period ranged from 12 to 102 months, with an average of 58 months. Short-term complications occurred in 1 patient (4.3%), who experienced a superficial skin infection that was treated with antibiotics. Long-term complications included four cases (17.3%) of non-union requiring revision surgery, along with two (8.6%) local recurrences that necessitated revision surgery, namely a modular endoprosthesis and above-knee amputation. There was 1 patient who underwent irrigation and debridement for a suspected infection, though the cultures came back negative. A total of 3 patients underwent lobectomy due to lung metastases. At the last follow-up, 19 patients (82.6%) were disease-free, 3 patients (13.4%) were alive with evidence of disease, and 1 patient (4%) was dead of disease. The average MSTS at the final follow-up was 23.16 ± 5.91.

## 4. Discussion

This study describes a knee joint-sparing technique that uses 3D-printed patient-specific instruments for the resection of bone tumors in the distal femur or proximal tibia, followed by reconstruction with either a customized allograft or a Ti64 implant with a lattice structure. Analysis showed that in 85% of cases, the patients still had a native knee joint regardless of being disease-free or having distant metastasis, which results in a better quality of life. To date, limb salvage surgery is the gold standard treatment for tumors surrounding the knee joint. To maintain a safe negative margin, the knee joint is usually sacrificed and a metallic modular endoprosthesis is used for joint arthroplasty and reconstruction. While this method has improved clinical outcomes, it still has relatively high complication rates, often leading to mechanical and aseptic loosening [9,10,11]. In cases where knee joint sparing is considered, the mere few mm between the joint surface and the distal margin of the tumor creates significant challenges for surgeons when operating free-hand. Although advances in imaging modalities such as MRI allow the surgeon to distinguish the exact extent of bone involvement during preoperative planning, it is still difficult to apply this knowledge practically in the operating theatre. However, creating a 3D digital model in addition to intraoperative PSIs for accurate osteotomies can compensate for the geometric challenges of joint-sparing surgery [16]. These knee-preserving tumor resections that preserve the normal joint anatomy result in better proprioception and better joint function after reconstruction [17,18].

The use of intraoperative PSIs is widely described in the field of orthopedic oncology for various applications; however, only a few studies demonstrate their application during knee-sparing surgeries. For example, Wang et al. utilized 3D-printed osteotomy guide plates to excise periacetabular malignant bone tumors in 11 patients, reporting shorter surgical times, reduced blood loss, and improved prosthetic matching [19]. Similarly, Li et al. reported the ability of 3D-printed guides to improve the excision of irregularly shaped tumors, such as giant invasive sacral schwannoma [20]. Wong et al. reported that 3D-printed PSIs significantly reduce intraoperative times and increase safety by helping to confine the oscillating saw to the planned resection plane, comparing it to both traditional methods and computer navigation methods [21]. Furthermore, Liu et al. reported that the use of 3D-printed guides in joint preservation for 12 patients with metaphyseal malignant bone tumors around the knee was cost-effective, and the reliability of the reconstruction in eliminating gaps between the implant and the host bone allowed patients to experience earlier partial weight barring and, ultimately, a return to good function [22].

Our results indicate that only four revision surgeries were required due to non-union of the allograft. Notably, since transitioning to the use of printed Ti64 implants, no complications have been observed. This suggests that future outcomes may continue to improve with the exclusive adoption of Ti64 cages. Additionally, we observed only two cases of local recurrence (8%), a promising result compared to the higher rates reported in the literature for tumor resection surgery. In the case of the patient who died from the disease, we believe the outcome was beyond surgical control. One year after surgery, the patient experienced a massive flare-up of the disease with both local recurrence and multiple metastases, which ultimately led to their death. These findings are encouraging, as knee-sparing tumor resections using 3D-printed PSIs can promote joint function and enhance proprioception by preserving native ligaments and maintaining normal joint surfaces. This suggests that knee-sparing surgery with printed cage reconstruction could become the gold standard for these procedures in the near future.

As part of the natural sequelae of knee-sparing surgeries, limb length discrepancy (LLD) is a common complication resulting from the disruption of normal growth plates. In our cohort, seven patients developed LLD, which was closely monitored throughout the follow-up period. Four of these patients required limb-lengthening procedures to address the discrepancy. LLD poses both functional and aesthetic challenges, affecting gait, balance, and overall quality of life. In pediatric patients or younger individuals with ongoing growth, the risk of developing LLD is particularly high, as surgical interventions may interfere with the natural growth potential of the limb. Despite efforts to mitigate this, it remains a persistent challenge in joint-sparing surgeries. Techniques such as guided growth, the use of expandable prostheses, or limb-lengthening procedures with external fixators or intramedullary nails are employed to correct LLD. However, these interventions carry their own risks and may not fully restore symmetry or function. This outcome highlights the need for further advancements in both surgical techniques and prosthetic design to better accommodate growth and minimize the development of LLD in patients undergoing joint-sparing surgeries. Continued research and innovation in this area are essential for improving long-term outcomes and the overall quality of life for these patients.

The benefits of 3D-printed PSIs described in the literature and reaffirmed by our experience have catalyzed both their increased incorporation into clinical practice and subsequent advances in the development process. Recently, Syed et al. conducted a systematic review of the literature, which, to date, is largely based on small cohorts and single-case studies regarding the manufacturing and use of PSIs, and has contributed to guidelines to optimize workflow and cost-effectiveness, as well as best practices for designs and materials [23]. Moreover, in their systematic review, Lal et al. found that 3D printing in general and customized PSIs in particular have found use in nearly all anatomical areas [24]. The work in our center contributes to this growing discourse. Our previous study [25] introduced the general workflow for utilizing 3D-printed patient-specific implants (PSIs) in long bone reconstructions, primarily focusing on cases where tumors were located in the femoral midshaft. That study demonstrated the feasibility of this approach but did not explore its application in cases requiring joint preservation. In contrast, the present study specifically investigates knee-sparing tumor resections, which present unique surgical challenges due to the proximity of the tumor to the joint surface. By targeting tumors in the distal femur and proximal tibia, this research expands upon our previous findings and highlights the advantages of a precise 3D-guided approach in maintaining native knee function, which has significant implications for patient quality of life and long-term functional recovery. Following resection, another challenge is the reconstruction of segmental bone defects. Various methods exist, including segmental tissue and prosthesis engineering methods, distraction osteogenesis, combined fibular autografts, vascularized fibular autografts, allografts, and irradiated autografts. Clinical studies have found that these methods often lead to complications such as significant blood loss, deep wound infection, prosthetic malfunction, and dislocation [26,27,28,29,30,31,32,33]. For example, in bony defect reconstruction following tumor resection, allografts have shown various advantages promoting graft–host integration and joint reconstruction [34,35,36]. However, complications, such as fractures stemming from revascularization of the allograft cortex or chemotherapy that induces an allogeneic immune response which leads to fractures, have ultimately required longer postoperative non-weightbearing to ensure graft union and protect reconstruction [37,38]. The Capanna procedure, combining a vascularized fibula with a massive allograft, has become the standard of care for limb salvage and long bone reconstruction since the authors reported a 93.5% long-term success rate in over 90 patients [39]. Despite these promising outcomes, various indications have been correlated with infection and non-union. For example, instances of non-union and infection were less frequent in femoral reconstructions (6%, 6%) compared to tibial reconstructions (10.5%, 8.5%), while allograft fractures were less prevalent in tibial (10.5%) than in femoral reconstructions (18%) [39]. While these techniques have become well-established practices, customized 3D-printed Ti64 implants to reconstruct segmental bone defects are rapidly becoming an optimal alternative as they allow early weight-bearing applications [15].

Lastly, compared to similar studies, our retrospective data present relatively long follow-up times. However, a major limitation of this study is the small sample size, which does not allow for a comparison of outcomes between different tumor characteristics and, thus, prevents us from reaching any absolute conclusions regarding further clinical use. Moreover, this study lacks a direct comparison group that uses the traditional free-hand joint-sacrificing approach. This was not included because the primary aim of this manuscript was to describe and evaluate a novel method for knee joint preservation in tumors. Additionally, comparing groups with significant differences in baseline characteristics and treatment protocols poses substantial technical pitfalls, making such comparisons statistically unreliable within the scope of this study. However, we acknowledge the importance of such comparisons and plan to address this in a future study focusing on the outcomes of patients treated with this method and those undergoing alternative procedures, including endoprostheses. Despite these limitations, this work represents the advantages made possible through 3D surgical planning of joint-sparing limb salvage in general and highlights knee-sparing surgery in particular as a safe reconstruction modality, without jeopardizing the oncological principles of bone tumor surgery.

## 5. Conclusions

To date, limb salvage surgery is the gold standard for treating lower limb sarcomas. However, when tumors are located near the knee joint, knee-sacrificing surgeries with modular prostheses are commonly performed. In this study, we demonstrate a 3D approach utilizing digital visualization and printed patient-specific instruments (PSIs) for knee-sparing bone tumor resections. Additionally, we propose a novel reconstruction method using customized printed Ti64 lattice implants. With the growing demand for personalized healthcare and the rapid advancements in digital 3D visualization and additive manufacturing, we believe that the future of orthopedic oncology lies in further refining these technologies. This progress will enable more precise, durable, and biologically integrated implants, ultimately leading to improved long-term outcomes for patients.

## Figures and Tables

**Table 1:** Demographics, diagnoses, and surgical outcomes.

Patient #	Age at Surgery	Sex	Pathology/Diagnosis	Site	Follow-Up (Months)	MSTS93 (%)
1	34	Female	Osteosarcoma	Femur	102	93.3
2	9	Male	Osteosarcoma	Femur	93	96.6
3	16	Male	Ewing	Tibia	89	86.6
4	6	Male	Ewing	Femur	86	N/A
5	44	Male	MFH of Bone	Tibia	85	26.6
6	20	Female	Osteosarcoma	Femur	83	76.6
7	12	Female	Ewing	Femur	78	96.6
8	17	Male	Osteosarcoma	Femur	76	90
9	18	Male	Osteosarcoma	Femur	76	83.3
10	9	Male	Osteosarcoma	Femur	66	43.3
11	20	Female	Chondrosarcoma	Femur	65	N/A
12	25	Male	Osteosarcoma	Femur	62	63.3
13	7	Female	Osteosarcoma	Femur	50	70
14	16	Female	Osteosarcoma	Femur	50	93.3
15	9	Male	Ewing	Tibia	47	N/A
16	34	Female	Atypical Cartilaginous Tumor	Femur	47	90
17	12	Male	Osteosarcoma	Femur	41	90
18	20	Male	Ewing	Tibia	37	93.3
19	17	Female	Osteosarcoma	Femur	35	N/A
20	14	Male	Osteosarcoma	Femur	21	50
21	14	Female	Osteosarcoma	Femur	21	80
22	56	Male	Chondrosarcoma	Tibia	13	86.6
23	59	Male	Chondrosarcoma	Femur	12	N/A

MFH—malignant fibrous histiocytoma. MSTS—Musculoskeletal Tumor Society scoring system. # Patient number 1–23.

**Table 2:** Surgical methods and outcomes.

Patient #	Resection	Reconstruction	Short-Term Complications	Long-Term Complications	LLD (cm)
1	Geographic	Allograft	None	None	
2	Intercalary	Vascularized Fibula and Allograft	None	None	6
3	Intercalary	Vascularized Fibula and Allograft	None	None	2
4	Intercalary	Allograft	None	None	8.5
5	Intercalary	Printed Cage Reconstruction	None	None	
6	Geographic	Allograft	None	Re-grafting due to non-union	
7	Geographic	Allograft	None	None	
8	Intercalary	Vascularized Fibula and Allograft	None	Re-grafting and re-fixation due to non-union	
9	Intercalary	Vascularized Fibula and Allograft	None	None	
10	Intercalary	Vascularized Fibula and Allograft	None	None	
11	Geographic	Allograft	None	Revision to megaprosthesis due to LR	
12	Geographic	Allograft	None	None	
13	Intercalary	Vascularized Fibula and Allograft	None	None	2
14	Intercalary	Vascularized Fibula and Allograft	Infection of soft tissues—antibiotics	None	1.5
15	Intercalary	Allograft	None	Capanna surgery due to allograft fracture	
16	Geographic	Allograft	None	None	
17	Intercalary	Vascularized Fibula and Allograft	None	Re-grafting and re-fixation due to non-union	3
18	Intercalary	Printed Cage Reconstruction	None	None	2
19	Intercalary	Printed Cage Reconstruction	None	I and D due to suspected infection	
20	Intercalary	Printed Cage Reconstruction	None	None	
21	Intercalary	Printed Cage Reconstruction	None	None	
22	Intercalary	Allograft	None	None	
23	Intercalary	Printed Cage Reconstruction	None	None	

I and D—irrigation and debridement. # Patient number 1–23.

**Table 3:** Margins, necrosis, and oncologic status.

Patient #	Margins	Tissue Margin	Necrosis	Oncologic Event	Oncologic Status
1	R0	Bone	N/A	None	NED
2	R0	Bone + Soft	87	None	NED
3	R1	Bone + Soft	40	None	NED
4	R1	N/A	100	None	NED
5	R0	Bone	95	LR + lung metastases	DOD
6	R1	Bone + Soft	N/A	None	NED
7	R0	Bone	100	None	NED
8	R0	Bone	99	Lobectomy due to lung metastases	NED
9	R0	Bone + Soft	96	None	NED
10	R0	Bone	100	AKA due to LR + lobectomy due to lung metastases	NED
11	R1	Bone + Soft	N/A	LR	NED
12	R1	Bone + Soft	78	MTS	AED
13	R1	Bone + Soft	91	Lung metastases	AED
14	R0	Bone + Soft	99	None	NED
15	R1	Bone + Soft	94	None	NED
16	R0	Bone + Soft	N/A	None	NED
17	R0	Bone + Suspected Soft	100	None	NED
18	R0	Bone + Soft	N/A	None	NED
19	R0	Bone	100	None	NED
20	R0	Bone	99	MTS	AED
21	R0	Bone + Soft	93	Lobectomy due to lung metastases	NED
22	R0	Bone	N/A	None	NED
23	R0	Bone	N/A	None	NED

NED—no evidence of disease. AED—alive with evidence of disease. DOD—death of disease. LR—local recurrence. AKA—above-knee amputation. # Patient number 1–23.

## Data Availability

Data are available upon request from the corresponding author.

## Abbreviations

The following abbreviations are used in this manuscript:

MFH	Malignant fibrous histiocytoma
I and D	Irrigation and debridement
NED	No evidence of disease
AED	Alive with evidence of disease
DOD	Death of disease
LR	Local recurrence
AKA	Above-knee amputation
